# Accurate Multiscale Simulation of Frictional Interfaces by Quantum Mechanics/Green’s Function Molecular Dynamics

**DOI:** 10.1021/acs.jctc.3c00295

**Published:** 2023-07-11

**Authors:** Seiji Kajita, Alberto Pacini, Gabriele Losi, Nobuaki Kikkawa, Maria Clelia Righi

**Affiliations:** † Toyota Central R&D Labs., Inc., 41-1, Yokomichi, Nagakute, Aichi 480-1192, Japan; ‡ Department of Physics and Astronomy, University of Bologna, 40127 Bologna, Italy

## Abstract

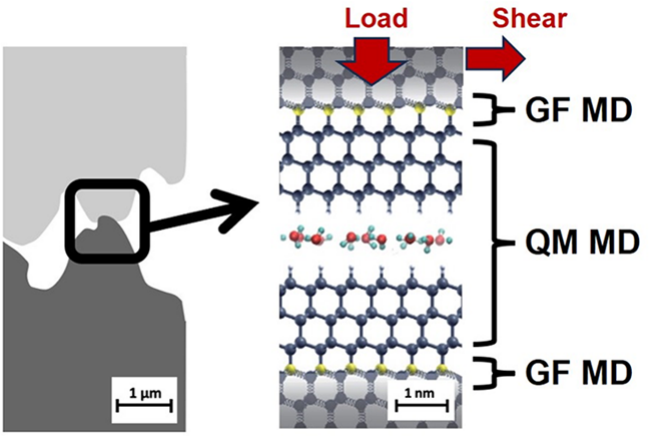

Understanding frictional phenomena is a fascinating fundamental problem with huge potential impact on energy saving. Such an understanding requires monitoring what happens at the sliding buried interface, which is almost inaccessible by experiments. Simulations represent powerful tools in this context, yet a methodological step forward is needed to fully capture the multiscale nature of the frictional phenomena. Here, we present a multiscale approach based on linked *ab initio* and Green’s function molecular dynamics, which is above the state-of-the-art techniques used in computational tribology as it allows for a realistic description of both the interfacial chemistry and energy dissipation due to bulk phonons in nonequilibrium conditions. By considering a technologically relevant system composed of two diamond surfaces with different degrees of passivation, we show that the presented method can be used not only for monitoring in real-time tribolochemical phenomena such as the tribologically induced surface graphitization and passivation effects but also for estimating realistic friction coefficients. This opens the way to *in silico* experiments of tribology to test materials to reduce friction prior to that in real labs.

## I Introduction

It is estimated that nearly one-third of the energy produced by fossil fuels to power vehicles is spent to overcome friction.^1^ Improved tribology technologies could dramatically reduce fuel consumption and CO_2_ emissions. However, with respect to other technologies based on materials, tribology is remarkably less advanced. The reason resides in the complexity and variety of the phenomena that occur at the sliding buried interface, which is difficult to monitor in real time by experiments. Simulations have a great potential to advance tribology, particularly those based on quantum mechanics, which is important for an accurate description of the chemical processes in conditions of enhanced reactivity. However, ab initio simulations as well as most of the atomistic methods nowadays used in tribology do not account for the energy dissipation by phonons.

At the atomistic level, frictional forces appear during the relative motion of two surfaces in contact because their interaction energy changes as a function of the relative lateral position, giving rise to a corrugated potential energy surface (PES). The energy for climbing the PES hills, provided by the external force, is partially lost in nonadiabatic hill descents via phonon excitation. It is clear from this simplified description of the frictional slip that the amount of dissipated energy is governed by two main factors: the PES corrugation and the phonon propagation into the bulks in contact. The PES corrugation is determined by the electronic properties of the interface,^2^ while phonon excitation and propagation depend on the elastic properties of the infinite bulks. The latter also determines how the applied mechanical stresses are transferred to the sliding interface. *In silico* experiments able to provide a quantitative estimate of the kinetic friction coefficient should then rely on a multiscale approach that includes both the electronic degrees of freedom at the interface and the vibrational degrees of freedom in the semi-infinite bulks.

Such a multiscale scheme is highly desirable also to accurately describe the activation mechanisms of tribochemical reactions, chemical processes involving environmental or lubricant molecules confined at the sliding buried interface. The rate of these processes is highly accelerated with respect to reactions thermally activated at the open surface in static conditions.^3,4^ For example, thin films known as “tribofilms” are synthesized *in situ* by mechanical rubbing additive molecules confined within micro-asperities contacts. These films are critical in preventing the cold sealing of nanoasperities and reducing the macroscopic friction and wear resistance of operating machinery parts. Mechanosynthesis, which exploits impact forces to efficiently produce functional compounds and medicines without the use of solvents^5,6^ is another important example where the control of the stress-assisted reactions is highly desirable.

Quantum mechanics (QM)-based molecular dynamics (MD) simulations can uncover elementary mechanisms of tribochemical/mechanochemical processes.^4,7^ Indeed, they have provided useful insight into several tribological phenomena such as the effects of humidity on the lubricity of carbon-based coatings,^8,9^ two-dimensional (2D) materials like graphene, and transition-metal dichalcogenides.^9−11^ Moreover, they allowed monitoring in real time the first stages of tribofilm formation from commercial additives^12−14^ or hydrocarbon molecules.^15^ However, these simulations cannot be used to quantify the kinetic friction coefficient because the limited thickness of the slabs which is typically used to model the solids in contact is too thin to contain the wavelength of the dissipated phonons. Indeed, several studies have reported that the energy dissipation associated with phonons, such as thermal conductivity and friction, are critically dependent on the size of the simulated systems.^16−19^ The phonon-energy dissipation occurs when a sliding solid resonates with low-frequency and long-wavelength modes of the counter solid.^20^ To describe such dissipative phonon modes in the simulations, one should directly involve the many solid atoms for long wavelengths and their slow dynamics, which cannot be approximated by naive methods such as velocity-proportional damping terms attached to the slab model.^17,18^

Green’s function (GF) molecular dynamics simulations and the related theory have been used to unleash the limitation of the limited system size.^17−26^ This approach projects the dynamical response of all of the degrees of freedom of the infinite solid atoms into a Green’s function, which can excite phonons of any long wavelengths that propagate toward the infinite bulk system without reflection. In other words, the phonon dissipation is implemented in a slab system, even though only the finite degrees of freedom are actually calculated. Convolutions of the Green’s function with applied forces represent effective forces of the surface atoms that take the infinite solid atoms into account in the dynamics. However, the calculation of the convolution is a critical computational bottleneck for the use of GF MD. A solution for such a problem has been recently proposed for general surfaces,^20^ based on the elegant analytical solution of the Green’s function using a fast convolution method.^27−30^ Therefore, the GF MD method can now be applied to large-scale simulations of realistic systems previously considered too computationally demanding. However, this framework is based on classical force fields, where the electronic degrees of freedom necessary to accurately describe the surface–surface interaction and the tribochemical processes are not considered.

To overcome this limitation, we propose a new multiscale approach that combines the strengths of the QM MD and GF MD. This is realized by a hybrid method that links the quantum-mechanical and GF molecular-mechanical parts of the system.^31^ The hybrid QMGF MD method can be used to obtain accurate quantitative estimates of the friction forces taking both interface chemistry and phonon dissipation into account. This can open the way to a novel understanding of tribological phenomena and allows for the execution of accurate tribochemistry experiments *in silico*.

The manuscript is organized as follows: Section II presents the theoretical framework (Sections II.I and II.II) and the computational implementation (Sections II.III–II.VI) of the GF MD method. An example of application is provided in Section III, focusing on the tribological properties of diamond as a function of surface hydrogenation and showing that the hybrid QMGF MD method is able to provide a quantitative estimation of the kinetic friction coefficients in agreement with experiments. Finally, the conclusions of this work are given in Section IV.

## II Methods

Here we describe the theoretical method and the numerical strategies implemented in the developed multiscale code. We start by reviewing the GF MD methodology shown in our previous work^20^ and then include details on the fast convolution and thermo-barostats. Finally, the hybrid add-remove method is presented, along with numerical strategies for stabilizing the dynamics in QMGF MD simulations.

### II.I Green’s Function of a One-Dimensional Chain

We begin with a semi-infinite one-dimensional chain as a simple example, which assists the readers in understanding the GF MD for general surfaces presented later. A chain composed of harmonic oscillators is considered, and the atoms are identical and connected with monotonic bonds modeled with springs of constant *K*. The equations of motion are
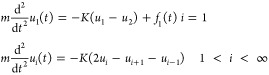
where *m* and *u*_*i*_ are the mass and displacement of the *i*th atom, respectively. The external force *f*_1_ is applied only on the edge atom *i* = 1. The displacement of the edge atom is mathematically written as the convolution form of the Green’s function *G*(32) with the force as
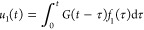
1The Laplace transformation of eq 1 is
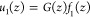
2where *z* is a coordinate in the complex space. It should be noted that *G*(*z*) is numerically more important than *G*(*t*) in GF MD with respect to both the fast convolution and the thermo-barostats methods explained later.

An atom, labeled by *i* = 0, is then coupled on top of the edge atom *i* = 1. This new atom becomes a surface atom under an external force, and its equation of motion after the Laplace transformation is
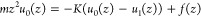
3where *f* is an external force on the new edge atom *i* = 0. Because *f*_1_ in eq 2 becomes counteracting force of the first term on the right-hand side of eq 3, eq 2 becomes
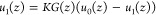
4By inserting eq 4 into eq 3 to eliminate *u*_1_, we obtain
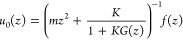
5

An important argument is that this addition of the *i* = 0 atom to a semi-infinite system in this way does not essentially change the original system due to its infinity. This invariant feature, called semi-infinite periodicity, simplifies the derivation of the Green’s function. Namely, because the periodicity tells that eq 2 is equivalent to eq 5, we can derive
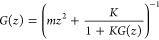
6eq 6 is readily solved as
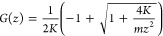
Derivation of *G*(*z*) without using the semi-infinite periodicity is more complicated as shown in ref (18). Semi-infinite periodicity is a key to generalize the method applicable to any surface system.

### II.II Green’s Function of a General Surface

**Figure 1:**
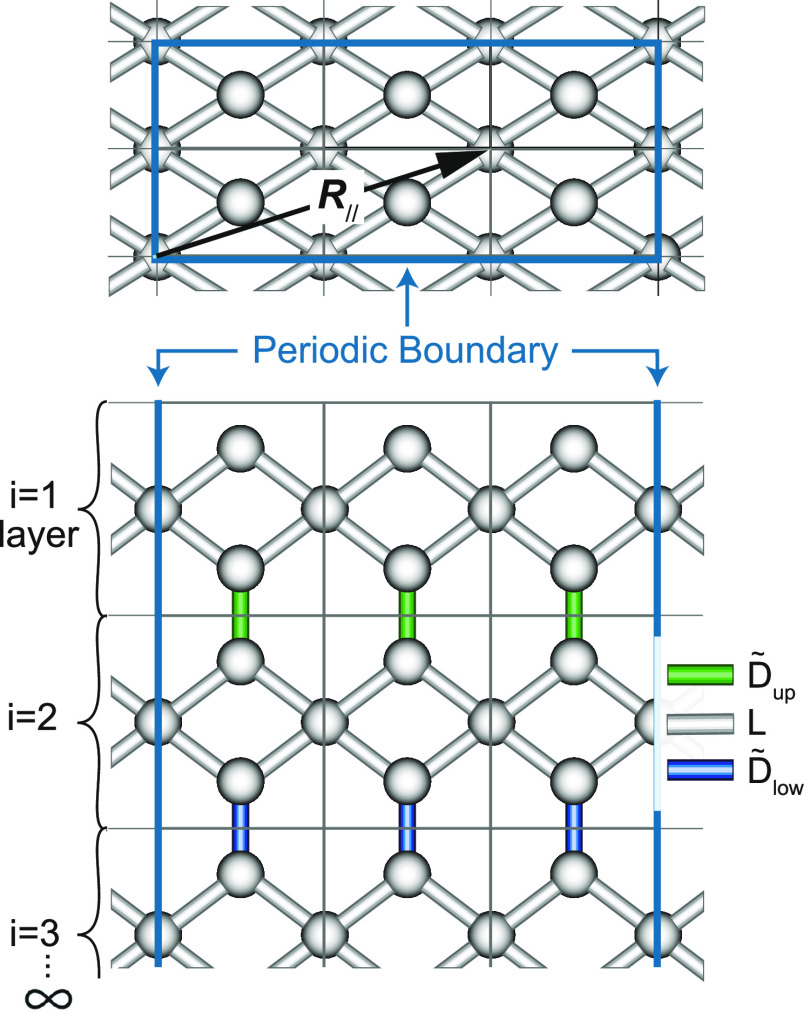
Schematic image of the semi-infinite surface system. The supercell consists of unit cells, and it repeats periodically along the surface lateral direction and infinitely along the direction normal to the surface. The layers are labeled by indices *i* that increase as going to the bulk direction. The colored bonds indicate interlayer terms *D̃*_up_ and *D̃*_low_ of the *i* = 2 layer; these quantities are used in Section II.II.

The strategy to derive the Green’s function of a general three-dimensional semi-infinite solid is the same as the one-dimensional chain. Let us consider a general crystalline surface as shown in Figure 1. We define a surface layer that is a set of unit cells laterally aligned in the periodic boundary conditions. Each layer is labeled with an index starting from the surface *i* = 1, 2, ···∞. This concatenation of the layers in the surface normal direction constitutes the semi-infinite solid. We write the equation of motion for the system as
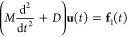
7where *M* is an atomic-mass diagonal matrix. The vector **u** represents the atomic displacements of the entire system, where **u** = (**u**_1_, **u**_2_, ···)^*T*^ and **u**_*i*_ is the displacement vector of the atoms in the *i*th layer. The external force vector **f**_1_ is applied only to the surface layer *i* = 1. The *D* matrix is referred to as an internal-force matrix that represents elastic constants of bonds for all of the atoms. Vectors, matrices, and scalars are indicated in bold, uppercase, and lowercase letters, respectively. We normalize eq 7 by the mass using an *N* = *M*^–1/2^ operator.
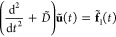
8where we define *D̃* = *NDN*, **ũ** = *N*^–1^**u** and **f̃**_1_ = *N***f**_1_.

A standard approach to include the periodic boundaries is the discrete Fourier transformation. A set of surface lattice vectors **R**_//_ points to the origins of the lateral positions of the constituent unit cells in the layer (see Figure 1, top). The discrete Fourier transformations of arbitrary vector **x** and matrix *X* of the layer are
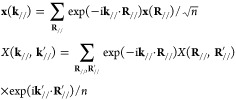
where **k**_//_ is the surface reciprocal vector of **R**_//_ and *n* is the number of unit cells in the layer. We use a matrix notation *X*(**k**_//_) when the matrix is diagonal with the **k**_//_ basis.

According to Bloch’s theorem, the internal-force matrix *D* is diagonal in the **k**_//_ basis due to the inherent periodicity of the system. In the initial conditions **u**(*t* = 0) = **u̇**(*t* = 0) = 0, eq 8 becomes
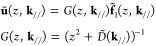
9after the discrete Fourier and Laplace transformations. Recalling the external force vector **f̃**_1_ is applied only on the surface layer *i* = 1, **ũ**_1_ is
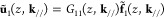
10where *G*_*ii*′_ is the corresponding element of *G* in the layer indices *i* and *i*′. In the **R**_//_ basis, eq 10 becomes
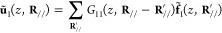
11

An additional layer *i* = 0 is piled up on the surface system by connection with the *i* = 1 layer. Before applying the semi-infinite periodicity, we decompose the internal-force matrix *D̃* into an intralayer term *L* that represents the bonds within the layer, and interlayer terms *D̃*_low_ ⊕ *D̃*_low_^′^ and *D̃*_up_ ⊕*D̃*_up_^′^, representing the bonds to the lower and upper layers, respectively (see Figure 1). Namely, the matrix representation of *D̃* in the layer index is
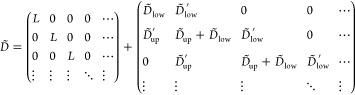


As in the previous subsection, we first write the equation of motion of the new layer
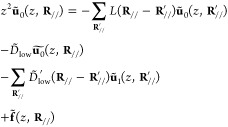
12The external force **f̃** is applied only on the *i* = 0 layer. Then, giving that **f̃**_1_ is a counteracting elastic force between **ũ**_0_ and **ũ**_1_, eq 11 becomes
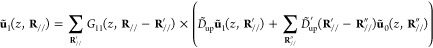
13The Fourier transformations of eqs 12 and 13 yield
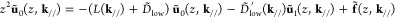
14
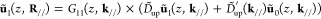
15Inserting eq 15 into eq 14 to erase **ũ**_1_, we obtain
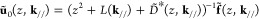
16where D̃* is an effective interlayer matrix defined by
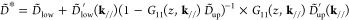


Finally, the semi-infinite periodicity promises that eqs 10 and 16 are equivalent because the new layer should respond to external forces in the entirely same manner as the original surface. We obtain an equation for the Green’s function as
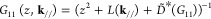
17The matrices *L* and *D̃* are numerically estimated by phonon calculations of the bulk system based on *ab initio* calculations. Eq 17 is solved by conventional Newton–Raphson algorithms.

### II.III Green’s Function Molecular Dynamics

The three-dimensional displacements of the semi-infinite surface layer atoms are described by a linear combination as
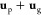
18where **u**_p_, **u**_g_ ∈ **R**^*N*×3^ are a particular solution and general solution^32^ of the equation of motion, respectively, and *N* is the number of the surface atoms in the unit cell. The solution **u**_p_ represents trajectories driven by an external force **f** applied on the surface layer, at initial conditions .

The general solution **u**_g_, on the other hand, is that without external force but in arbitrary initial conditions . Notably, **u**_g_ can represent the thermostat and barostat of the system when their statistic features are related to the Green’s function.

This subsection provides numerical recipes on how to compute **u**_p_ and **u**_g_.

#### II.III.I Particular Solution and Convolution

By using the Green’s function in eq 17, the equation of motion of **u**_p_ can be written as
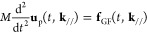
19
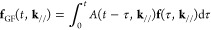
20

where the Laplace transformed *A* is defined as *A*(*z*, **k**_//_) = *z*^2^*N*^–1^*G*_11_(*z*, **k**_//_)*N* and **f** is an applied force on the surface layer. The reduced force **f**_GF_ has a convolution form, which becomes a computational bottleneck if discrete integral algorithms are used. The integral range grows as time *t* increases and the entire history of the force trajectory should be saved in memory. Indeed, the simulation time and memory allocation are proportional to *O*(*t*^2^) and *O*(*t*), respectively.

**Figure 2:**
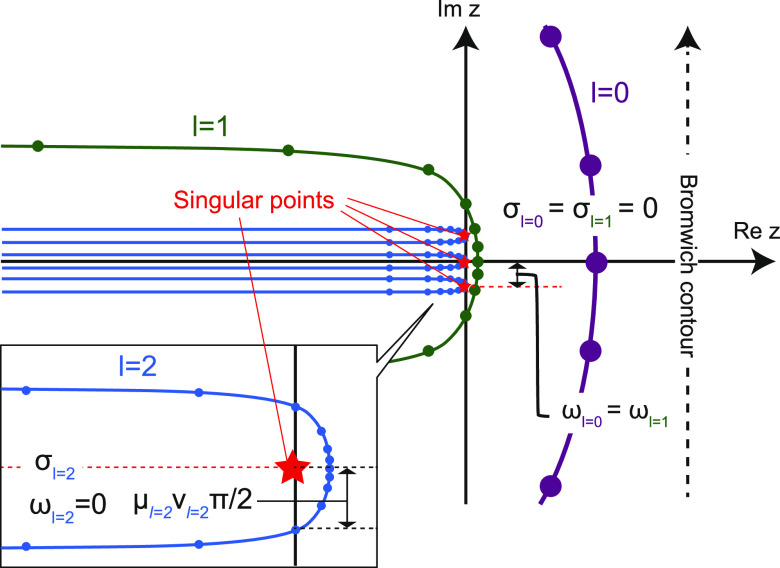
Schematic of integral paths of the mTILT. The paths labeled by index *l* are used in the time range *I*_*l*_ in eq 21. The parameter σ_*l*_ indicates a center of the contour in the imaginary axis, which is set with respect to imaginary parts of the singular points. The ω_*l*_ is assigned to the distance of the imaginary parts between the contour center σ_*l*_ and the farthest singular point. The width of the *l* path is the distance of the imaginary parts between σ_*l*_ and a cross section of the contour with the imaginary axis, which is equivalent to μ_*l*_ ν_*l*_ π/2.

A fast convolution based on modified Talbot’s inverse Laplace transformation (mTILT)^27−30^ reduces this notorious computational costs into *O*(*t*log(*t*)) for the simulation time and *O*(log(*t*)) for memory allocation. The conventional inverse Laplace transformation of an arbitrary function *X*(*z*) is defined by
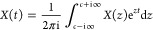
where the constant *c* is a real number larger than zero. This integral path is called Bromwich contour. The idea of mTILT is that the Bromwich contour is bent in such a way as to encircle singular points of *X*(*z*) on the imaginary axis, as shown in Figure 2. Coordinates of the singular points of the Green’s function are identified by a line search of *G*_11_(*z* = iω′, **k**_//_), where ω′ is a real number variable. The mTILT divides the time range [0, *T*] into a set of time ranges *I*_*l*_ as follows
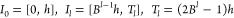
21where *h* is a time step and *l* = 1, 2, ···, *L*. The integer *L* satisfies (2*B*^*L*^ – 1) *h* ≥ *T* and *B* is an arbitrary integer greater than 1. The integral path used in *I*_*l*_ is defined as
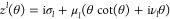
where the geometry parameters are μ_*l*_ = μ_0_/*T*_*l*_, μ_0_ = 8, ν_*l*_ = ν_0_(1 + ω_*l*_/β), ν_0_ = 0.6, and β = π μ_*l*_ ν_0_/2.

For example, Figure 2 illustrates the shapes of the paths *z*^*l*^. The widths of *l* = 0 and *l* = 1 paths are large enough to enclose all of the three singular points. As *l* increases, the width of the path μ_*l*_ν_*l*_π/2 decreases because μ_*l*_ decreases. The number of *l* = 2 paths, in this example, becomes three, and each path encloses each of the singular points.

In this manner, the mTILT designs the integral paths to secure high numerical accuracy of the inverse Laplace transformation, depending on the time interval *I*_*l*_. Namely, when *t* ∈ *I*_*l*_, the inverse Laplace transformation is
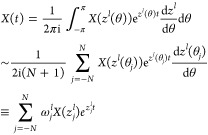
22where a trapezoidal rule in the integral range [−π, π] is used with discretization θ_*j*_ = *j* π/*N* + 1, *j* = −(*N* + 1), ···, *N* + 1. We defined  and *z*_*j*_^*l*^ = *z*^*l*^(θ_*j*_). For notation simplicity, eq 22, which represents the single path embracing all of the singularity, will be used in the following. In the case of the plural paths as *l* = 2 in Figure 2, contributions calculated by eq 22 are merely summed up.

Then, the mTILT is applied to the convolution task in eq 20. A range of the simulation time [0,*T*] is divided according to eq 21 in the convolution routine. Namely, when , the convolution is approximated as
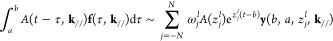
23where **y**(*b*, *a*, *z*,**k**_//_) = ∫_*a*_^*b*^ e^*z*(*b*–τ)^**f**(τ,**k**_//_)dτ. Here, we omit **k**_//_ variable unless the context needs it explicitly. The quantity **y**(*b*, *a*, *z*) is known to be a solution of the following differential equation at *t* = *b*
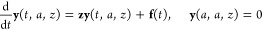
24We then approximate **y** by time-discretized **f**(*t*_*k*_). The time interval [*a*, *b*] is split into a sequence of partial intervals [*a* + *t*_*k*_, *a* + *t*_*k*+1_], where *t*_*k*_ = *k* × *h* and *k* = 0, ···, *n* = (*b* – *a*)/*h*.

In *t* ∈ [*a* + *t*_*k*_, *a* + *t*_*k*+1_] ⊂ *I*_*l*_, eq 24 is expressed as
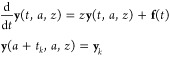


An exact solution of this equation is
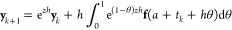
We apply a linear approximation **f**(*a* + *t*_*k*_ + *h*θ) ∼ θ**f**_*k*+1_ + (1 – θ)**f**_*k*_, where **f**_*k*_ = **f**(*a* + *t*_*k*_). As a result, the approximated solution **y**_*k*_^′^ can be obtained via a recursive expression with respect to the index *k*
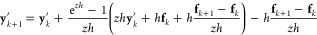
25

Since the mathematical components have been prepared, we now describe the fast convolution integral. Denoting *t* = *t*_*n*+1_, we divide the range of convolution into two regions as
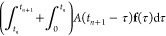
26The modified Talbot path of *I*_0_ calculates the first term as
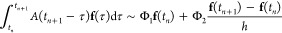
27where
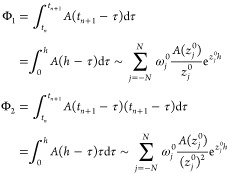
The second term of eq 26 is decomposed into contributions of the intervals *I*_*l*=1,2,_···_,*L*–1_, where *L* is the minimum integer that satisfies *t*_*n*+1_ < 2*B*^*L*^*h*. We define τ_0_ = *t*_*n*_, τ_*L*_ = 0, and τ_*l*_ = *q*_*l*_*B*^*L*^*h* if *l* ≠ 0 nor *L*. An integer *q*_*l*_ ≥ 1 is determined so as to satisfy
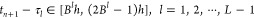
The time range is divided as [0, *t*_*n*_] = ∪_*l*=0_[τ_*l*_, τ_*l*–1_]. Therefore, by using the approximations in eqs 23 and 25, we derive
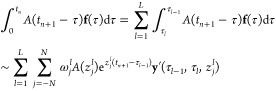
28In short, the convolution term is calculated by the sum of eqs 27 and 28, along with eq 25 which is used for efficient calculation of the term **y**′. Then, because the reduced force is obtained, the motion equation eq 19 is numerically solved to simulate the trajectory. A simple example of specific steps to show the **y**′ updating is given in the Supporting Information.

#### II.III.II General Solution and Thermo-Barostats

We consider the general solution **u**_g_ in eq 18. Let us use **u** instead of **u**_g_ because of notation simplicity. The mass-normalized equation of motion for the whole system in the **k**_//_ space is
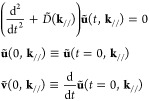
29where **ũ** = (**ũ**_1_, **ũ**_2_, ···)^*T*^ in the layer index representation. The Laplace transformation of eq 29 yields
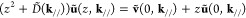
Using eq 9, we can describe the general solution in the Green’s function framework, as
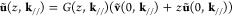
30

The initial condition includes all of the displacements and velocities in the semi-infinite system. Obviously, there is an infinitely large number of possible configurations of the initial conditions. A reasonable policy to select a physically meaningful one is to consider a thermostat. The semi-infinite system is assumed to be located at a temperature *T*, and the constituent atoms move according to the thermal fluctuation. Let this general solution be denoted by **ũ**_*T*_. We modify eq 30 by using notations *S*(*z*) ≡ *zG*(*z*) and **ṽ**_*T*_(*z*, **k**_//_) ≡ *z***ũ**_*T*_(*z*, **k**_//_), and apply the inverse Laplace transformation.
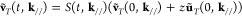
31Here, we use the law of equipartition of energy
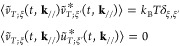
where ξ refers to the components of the atomic coordinates (*x*, *y*, *z*) and * is the complex conjugate. The bracket represents the ensemble averaging operator. By applying the equipartition law, eq 31 becomes
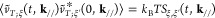
32that is called the fluctuation-dissipation theorem. This relation tells that an auto-correlation of the general solution of the velocity should be equivalent to the Green’s function.

Another useful general solution represents normal and shear stresses. A semi-infinite system is located at 0 K temperature under a uniform stress applied to the surface **f**(*t*, **k**_//_) = −δ_**k**_//_,**0**_**f**_*s*_. The velocity solution of this system is
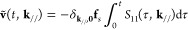
where δ is the Kronecker delta. As time *t* goes, the semi-infinite system deforms by the applied stress. In the limit of *t* → ∞, the deformation eventually stops at a configuration that balances the applied stress and elastic force, namely, **ṽ**(*t* → ∞, **k**_//_) = 0. At this stage, the elastic energy stored by the deformation produces a general solution **ṽ**_*S*_(**k**_//_) that satisfies
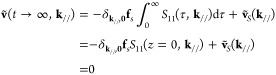
we obtain
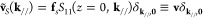
33Interestingly, this equation indicates that the applied stress is proportional to the constant velocity term. Note that a general solution from initial conditions of constant velocity, which is **ũ**(0, **k**_//_) = 0, **ṽ**(0, **k**_//_) = **v** δ_**k**_//_,**0**_ is equivalent to eq 33. Namely, the constant shear stress becomes the same as the initial condition in which we start the dynamics by giving the constant velocity to the semi-infinite solid system.

In short, by adding d**ũ**_*g*_/d*t* = **ṽ**_*T*_ + **ṽ**_*S*_ to the trajectory of the surface layer, we can control the temperature, normal stress, and sliding velocity of the semi-infinite system.

#### II.III.III Numerical Treatment of Thermostat

We show a numerical recipe to generate random velocity which holds the fluctuation-dissipation theorem in eq 32. An algorithm proposed by Berkowitz^33^ is used. By assuming that *ṽ*_*T*;ξ_(*t*, **k**_//_) is periodic in an enough long period *P*, the Fourier series expansion yields
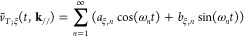
34where ω_*n*_ = 2π*n*/*P*. The random variables *a*_ξ,*n*_ and *b*_ξ,*n*_ are assumed to be independent. By inserting eq 34 on the left-hand side of eq 32, it becomes
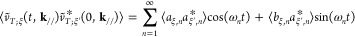
35We extend the domain *t* ≥ 0 of *S*_ξ,ξ′_(*t*, **k**_//_) to ∞ ≥ *t* ≥ −∞ by using *S*_ξ,ξ′_(|*t*|, **k**_//_). The right-hand side of eq 32 is modified as
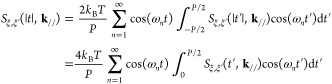
Because of the assumption that *P* is enough large, we can use the cosine transformation . Therefore
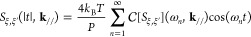
36Inserting eqs 35 and 36 into eq 32, we obtain relations of the random variables required by the fluctuation-dissipation theorem, as
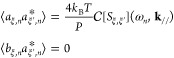
because randomness of the *ṽ*_*T*;ξ_(*t*, **k**_//_), the ensemble average of the magnitudes of *a*_ξ,*n*_ and *b*_ξ,*n*_ are equivalent: ⟨*a*_ξ,*n*_*a*_ξ′,n_^*^ ⟩= ⟨*b*_ξ, *n*_*b*_ξ′, n_^*^ ⟩.

Then, we construct a covariance matrix Σ to generate the random variables *a* and *b*.
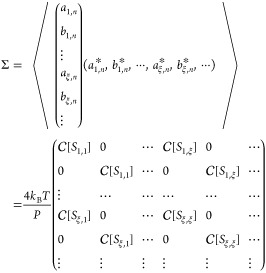
37This matrix is the Hermite matrix, given . A stochastic vector **x** = (*A*_1,*n*_, *B*_1,*n*_, ···, *A*_ξ,*n*_, *B*_ξ,*n*_, ···) that satisfies eq 37 can be generated by considering a multivariable Gauss distribution  with the covariance matrix Σ.
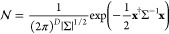
38where the number of elements of **x** is 2*D*. We define a matrix *U* that diagonalizes Σ and its eigenvalue matrix λ. The variable **x**^†^ Σ^–1^**x** is transformed as
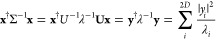
39where **y** = *U***x**. Inserting eq 39 into eq 38, the Gauss distribution becomes
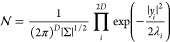
40Equation 40 indicates that  is expressed by the products of independent Gaussians that have no correlation between the variables. Therefore, Box–Muller method can be used to generate stochastic variables regulated by the Gauss distribution. The variable *y*_*i*_ is calculated by
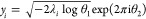
where θ_1_ and θ_2_ are uniform random variables ranging from 0.0 to 1.0. Then, converting **x** = *U*^–1^**y**, we obtain the thermal velocity terms *ṽ*_*T*;ξ_(*t*, **k**_//_) via eq 34. In this study, we use *P* = 2^19^*h*.

### II.IV Coupling the QM and GF Systems

#### II.IV.I Add-Remove Method

**Figure 3:**
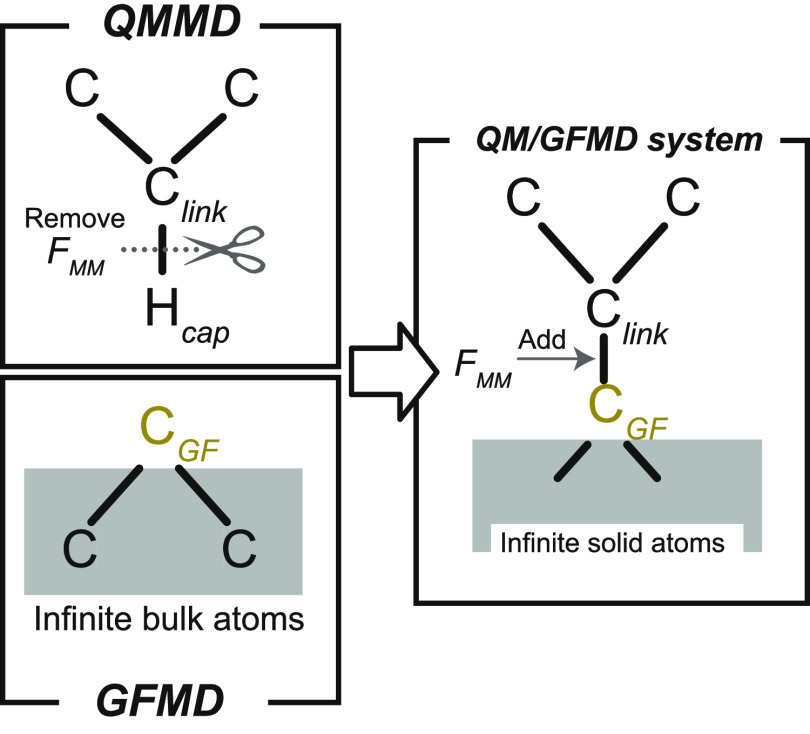
Schematic representation of the add-remove method in a diamond surface of the QMGF system. The force *F*_MM_ indicates a classical force field of the corresponding bond.

To couple two systems of different scales, their junction should be bridged smoothly.^31,34−36^ This study uses an add-remove method, which is one of the hybrid schemes for solids.^31^ Hydrogens are often used to cap the boundaries of a QM system to stabilize the unsaturated edge atoms, while mechanical contributions such as forces from the artificial cap atoms are eliminated because they should not be present in the junction.^31,36^Figure 3 shows an outline of the method in the diamond slab, where H_cap_ and C_link_ denote the cap hydrogens and linked carbons, respectively. A surface carbon generated by GF MD is indicated by C_GF_.

The add-remove method works as follows:

1. H_cap_–C_link_ bonds are removed by subtracting the corresponding classical force fields.
2. The QM and GF systems are connected with a classical C_link_–C_GF_ bond.
3. The positions of H_cap_ are located along the projection of the straight line connecting C_link_ and C_GF_. The bond length of H_cap_–C_link_ is fixed at its equilibrium distance.
4. The forces are corrected due to the constraint of the H_cap_ position.

We describe the details of points 3 and 4. The constrained position of the cap hydrogen **r**_cap_ are
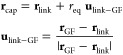
41where **r**_link_ and **r**_GF_ are the positions of C_link_ and C_GF_, respectively. The symbol *r*_eq_ indicates the length of bond H_cap_–C_link_ fixed at its equilibrium distance. Due to the **r**_cap_ constraint, the forces should be corrected. We consider a Hamiltonian of the whole system  including contributions of the add-remove method.
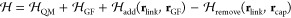
where  and  indicate the original Hamiltonians of the QM and semi-infinite harmonic oscillator systems, respectively (see Section III for a description of the system). The addition and removal operations in Figure 3 are represented by  and , which come from the classical interactions of C_link_–C_GF_ and H_cap_–C_link_, respectively. Given the constrained **r**_cap_(**r**_link_, **r**_GF_) in eq 41, the forces acting on C_GF_ and C_link_ atoms are
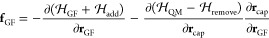
42
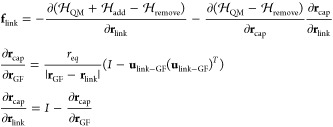
43where *I* is the unit matrix. Note that **u** (**u**)^*T*^ indicates the dyadic product. The first terms on the right-hand sides of eqs 42 and 43 consist of interaction forces obtained by GF MD in eq 20 and QM MD simulations along with the add-remove classical force terms, respectively. The second terms in eqs 42 and 43 come from the constraint of eq 41. These force terms become zero if the classical force of H_cap_–C_link_ completely agrees with the QM bond. However, because the classical model cannot reproduce the quantum method perfectly, these constraint-force corrections should be included to keep the energy conservation law.

#### II.IV.II Refresh Strategy

Simulations of sliding friction typically require several hundred thousand steps. As shown in eq 20, the convolution of the GF MD increases its integral time range as time evolves. This fact induces an accumulation of the integral errors in such a long simulation, leading to inaccurate dynamics and overall instability of the GF MD simulation. This subsection provides a remedy for this issue.

**Figure 4:**
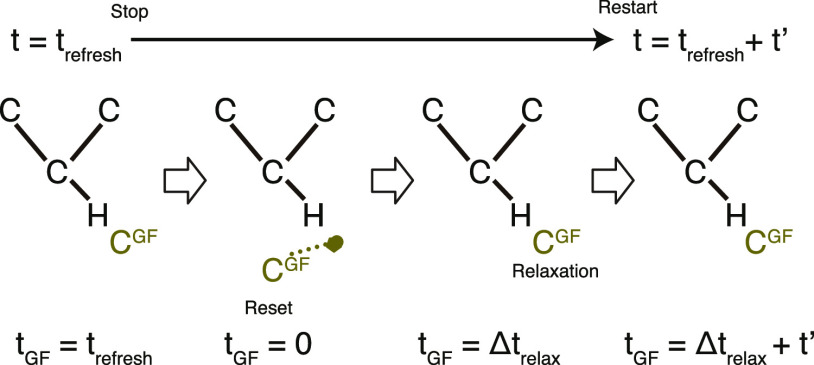
Schematic of the refresh treatment process. The dotted line indicates a spring that is used for the relaxation step presented in the main text.

Given that the error comes from the extension of the integral region, an idea would be to reset the convolution before the error cannot be ignored anymore. Figure 4 presents an outline of this treatment, which we call ”refresh”. Two clocks *t* and *t*_GF_ are prepared for QM and GF MD systems, respectively. The two clocks advance exactly in the same manner at the start of the QMGF MD simulation. Once they arrive at a user-defined *t*_refresh_, at which the GF MD numerical error is considered critical, the positions **r**_GF_(*t*_refresh_) are saved as anchors in the memory. At this point, the *t* clock stops, but only the *t*_GF_ clock is reset to zero to make the integral range of the convolution zero. The positions **r**_GF_ are connected to the anchored positions with specific springs to be arranged to their initial positions with respect to **r**_link_. When we start the *t*_GF_ clock, but still keep the t clock stopped, the springs pull the C_GF_ in such a way that **r**_GF_ returns to the anchored positions as a result of the relaxation. After a certain relaxation time Δ*t*_relax_, the springs are removed and the *t* clock starts to run together with *t*_GF_.

**Figure 5:**
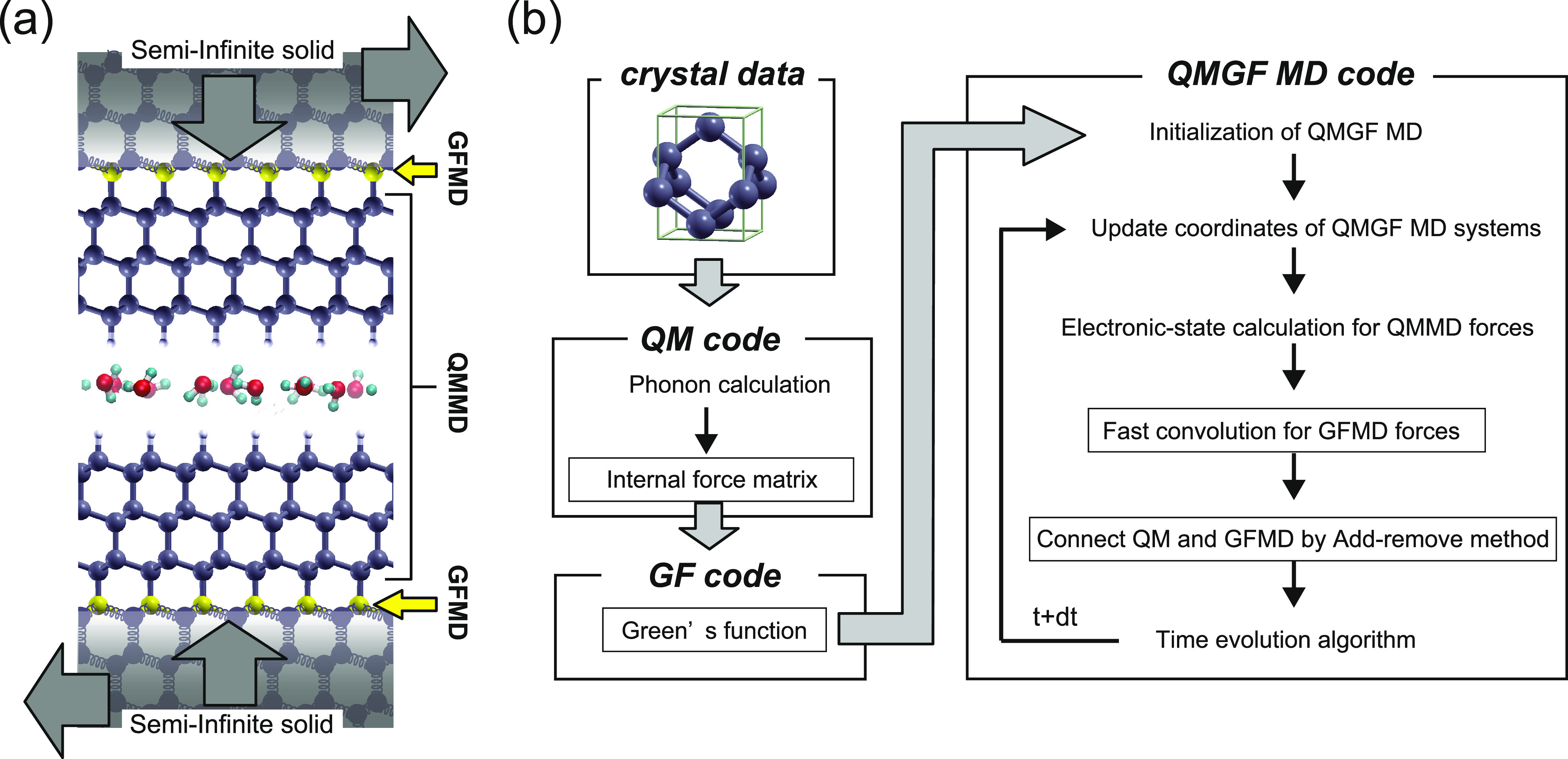
Representation of a frictional interface described by the QMGF MD hybrid scheme. The GF MD atoms at the boundary of the QM region are colored in yellow, and the semi-infinite bulks are represented as coupled harmonic oscillators (a). The workflow of the developed QMGF MD program by linking an open-source *ab initio* code^37^ to the in-house developed GF MD code (b).

By iterating this refresh every time *t*_GF_ = *t*_refresh_, we can perform long and stable GF MD simulations. This treatment, however, provides artificial effects to C_link_ when the *t* clock restarts because the velocities of C_GF_ are lost as a consequence of the relaxation. Nonetheless, because C_link_ are the junction atoms of the hybrid system in the QM bulk region (see Figure 5a), this error can be regarded as a perturbation that does not affect surface phenomena if the QM slab model consists of several atomic layers.

### II.V Computational Details

The internal-force matrix *D* is calculated by static *ab initio* calculations of the diamond bulk, based on density functional theory (DFT) and a DFT linear-response approach to phonons calculation,^38^ performed with the pw.x and ph.x solvers from the Quantum Espresso package.^37,39,40^ The Perdew–Burke–Ernzerhof generalized gradient approximation is used for the exchange-correlation functional.^41^ Electronic wave functions are expanded on a plane-wave basis set with a cutoff energy of 25 Ry, and ionic species are described by ultra-soft pseudopotentials.^42^ The matrix is approximated so that it only contains elements related to the nearest-neighbor interactions. The off-diagonal elements of the directional indices are also eliminated for the sake of numerical simplicity.

For the add-remove method, the classical force field of the C_link_–C_GF_ bond is set at the value of the corresponding element of the internal-force matrix. The C_link_–H_cap_ spring constant is estimated from *ab initio* static calculations performed on a fully H-terminated 2 × 1 (111) diamond slab of 12 atomic layers. The estimated spring constant of the surface normal direction is 0.2575 Ht/bohr, while for the surface lateral direction is 0.0365 Ht/bohr. The stable bond length of C_link_–H_cap_ is *r*_eq_ = 2.1043 bohr.

We implemented the QMGF MD hybrid method into the Car-Parrinello solver cp.x. The time development of cp.x is solved by the Verlet method, which does not use the velocities of atoms explicitly. On the other hand, the GF MD uses the general solutions to impose the temperature and stress by adding the velocity corrections **v**_*T*_ and **v**_*S*_. In order to merge the velocity correction into the QM MD algorithm, we used the leap-frog method that explicitly leverages the velocity term but is compatible to the Verlet method, as follows
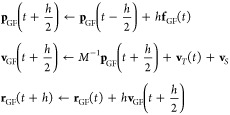
where **p**_GF_, **v**_GF_, and **r**_GF_ are momentum, velocity, and position vectors of the GF MD atoms, respectively. The time step is set to *h* = 0.1 fs, and the reduced force **f**_GF_ is calculated by eq 20. The parameters of mTILT are *B* = 11 and *N* = 60, which is equivalent to the 121 integral points in the contour. The singular points of the Green’s function are searched by evaluating its first and second derivatives on the imaginary axis. In the refresh treatment, we use *t*_refresh_ = 50,000 h, the anchor spring constant is 0.05 Ht/bohr for all of the *x*, *y*, and *z* directions, and the relax time is 2000 h. Temperature is set to 300 K by the thermostat of the GF MD method.

The QM ions are thermalized by applying a Nosé–Hoover thermostat with a frequency of 80 Thz and imposing an average electronic kinetic energy of 0.25 atomic units on the electron degrees of freedom. The electronic mass and the time step of the molecular dynamics are selected to be 100 and 4 atomic units, respectively. At the beginning of our dynamic simulations, the CP solver is employed to obtain the ground state energy of the electronic wave functions with the steepest descent algorithm. Subsequently, the hybrid QMGF MD code is used to carry out the dynamic simulation. The computational parameters adopted for the CP scheme have been carefully selected to achieve good accordance between the temperatures of the QM and GF atoms during the dynamics of the system under study.

### II.VI Summary of the QMGF Method

A pictorial representation of the hybrid QMGF MD scheme and its application to a prototypical tribochemistry system is offered in Figure 5. The chemically active part of the system consists of two surfaces in contact and some molecules eventually confined between them (Figure 5a). The inclusion of the electronic degrees of freedom is necessary to capture quantum effects, such as the Pauli repulsion at the short distances imposed by the applied load and the enhanced chemical reactivity of confined species, which deeply affect the tribological behavior. The two semi-infinite bulks are described by a collection of an infinite number of harmonic oscillators of first-principles-derived spring constants. Their effect is fully taken into account by the surface atoms indicated in yellow. The basic idea of GF MD is, in fact, that all of the internal modes of an elastic solid can be integrated out and substituted by effective interactions.^24,43^ In this way, only the trajectories of the quantum atoms and the surface atoms treated by the GF MD are needed, and no other bulk atoms are needed to be included in the simulation. The workflow of the QMGF MD method is shown in Figure 5b. The model for the bulk crystal is constructed, and static first-principles calculations are used to obtain the force matrix, which is used to calculate the Green’s function. The QM and GF systems are finally coupled via an add-remove scheme.^31^

## III Results for Diamond Interfaces

**Figure 6:**
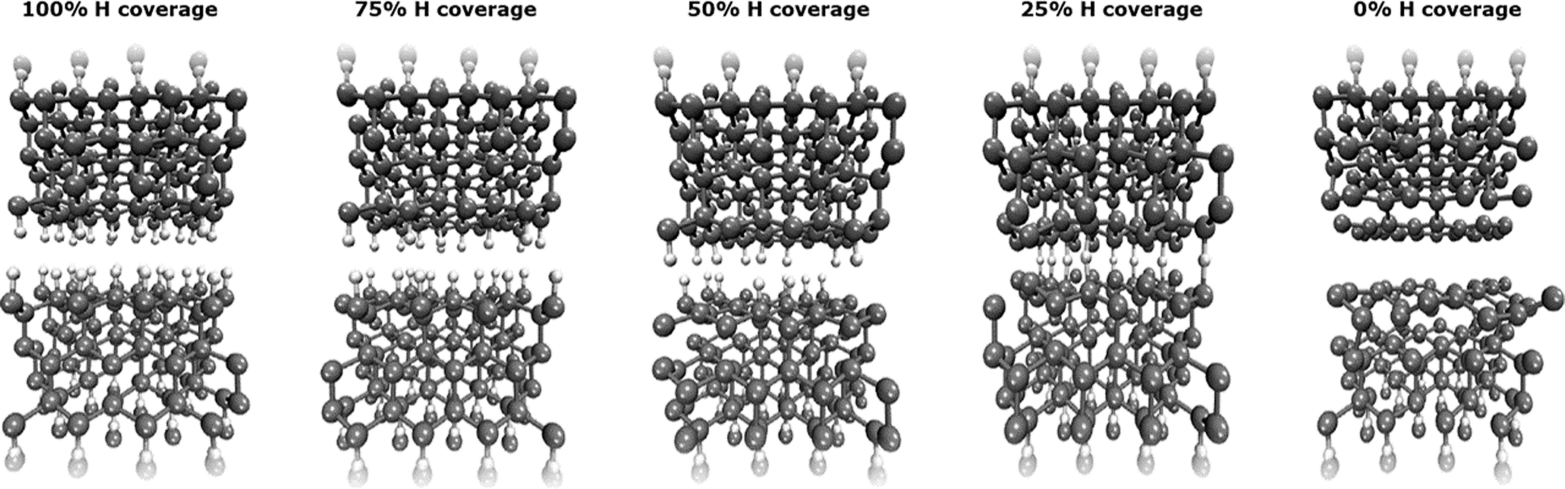
Lateral view of the diamond-on-diamond systems after 10 ps of sliding motion. The surface energy increases with the number of unsaturated carbon atoms, producing higher adhesion and smaller separation. When hydrogen is completely removed, a partial graphitization of the interface can be recognized, and the interfacial separation becomes similar to the interlayer distance of graphite (3.3 Å).

We employed our QMGF MD solver to study the sliding interface between two diamond crystals and quantitatively estimate the friction coefficient considering different concentrations of H atoms on the two mated surfaces. We focused our attention on the C(111) surface, the most accessible cleavage plane of diamond, and modeled the diamond-diamond interface by adopting a supercell with (4 × 2) in-plane size, containing two-faced slabs, each constituted of three bilayers of carbon atoms. The slabs are externally passivated by hydrogen atoms and the GF atoms are linked to these capping atoms, as described in the Section II. The interfacial region, where the two surfaces are faced, contains hydrogen atoms in different concentrations and randomly distributed. In Figure 6, a lateral view of all of the considered systems after 10 ps of sliding is reported.

We performed molecular dynamics simulations at a temperature of 300 K with an external load of 5 GPa for a time interval of ∼50 ps. To generate the sliding motion, we applied shear stresses of 1 GPa along the *x* direction by applying external lateral forces in opposite directions on the GF MD atoms of each slab. As described in Section II.III.II, the surface slabs slide against each other at constant velocity if there is no friction force, because the condition of the constant shear stress is equivalent to a situation where we start the friction test by imposing a relative velocity on the semi-infinite solids.

### III.I Effects of Interfacial Adhesion on Kinetic Friction

Three values of the H-coverage, θ, turned out to be high enough to enable the sliding motion under the effects of the applied lateral forces. Instead, the other coverages were too low to prevent chemical bonds from forming across the interface, which impeded the lateral displacement.

**Figure 7:**
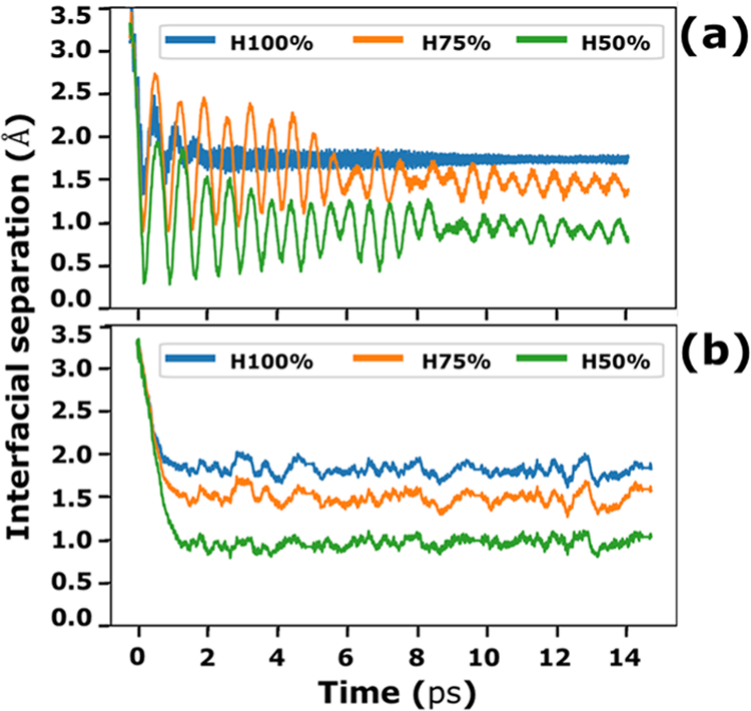
Interfacial separation for the 100, 75, and 50% passivated systems during the QM MD (a) and QMGF MD (b) simulation of sliding. The surface separation is calculated by considering the *z* coordinates of the hydrogen atoms.

The results in Table 1 highlight the effect of surface passivation on kinetic friction. A decrease in hydrogen coverage always results in a friction increase with a corresponding reduction in sliding velocity and average slab separation, also shown in Figure 7a. This behavior can be explained in terms of the chemical reactivity of the facing diamond surfaces. When H atoms are removed from the diamond surface, the terminal C atoms expose dangling bonds, which are very reactive. The dangling bonds of two surfaces in contact interact and cause a significant increase in the adhesive friction of the system. The calculated friction coefficients are in agreement with diamond-on-diamond experiments in an air environment, where μ_*k*_ ranges between 0.01 and 0.1.^44−46^ This extremely low friction has been detected for different surfaces of diamonds, e.g., the (100),^45,47−49^ (110)^47^ and also for nanocrystalline diamond films and diamond-like carbon (DLC) employed as coatings in technological applications.^46,50^ Since our simulations are representative of a single asperity contact, the most relevant data to compare with is the friction coefficient of 0.05 measured for a diamond tip sliding on the (111) diamond surface, which was obtained by dividing the measured friction force by the applied load,^49^ as the coefficients reported in Table 1. The study of the dependence of the friction force on load through the QMGF MD method will be the subject of further investigations.

**Table 1:** Results of the QMGF MD Simulations^a^20

Quantities derived from the sliding dynamics				
θ	⟨*d*_*C*_⟩	⟨*v*_*x*_⟩	⟨*F*_*x*_^*k*^/*A*⟩	μ_*k*_
100%	4.04	51	0.15	0.03
75%	3.72	51	0.21	0.05
50%	3.25	48	0.27	0.06

a For each hydrogen coverage, θ, the averages of the surface separation ⟨*d_C_*⟩ (Å), sliding velocity ⟨*v_x_*⟩ (m/s), kinetic friction force per unit area ⟨*F_x_^k^*/A⟩ (GPa), and the kinetic friction coefficient μ*_k_* are calculated. ⟨ *F_x_^k^* ⟩ is calculated as the time average of the interfacial forces acting on the GF atoms along the *x* direction. These are the forces appearing in the convolution integral in eq 20. The kinetic friction coefficient is calculated as the ratio between ⟨*F*_x_^k^⟩ and the applied load on the GF atoms.

In particular, for a diamond tip sliding on the (111) diamond face, the friction coefficient obtained as the ratio of the measured friction force and the applied load is 0.05,^49^ which is almost constant along any possible sliding direction and independent from the applied load. Since our system may be representative of a his experimental result are in agreement with the values extracted from our simulations. It should be kept in mind that the considered interfaces are commensurate, and the magnitude of calculated friction coefficients can change for the incommensurate case.^51^ However, we do not expect that the trend observed by changing the degree of interface passivation will change. The choice of considering commensurate surfaces can be justified considering that our system can represent a nano-asperity-contact, such as an AFM tip, where the tip atoms are expected to conform to the substrate because of the small area of contact, as shown by simulations for silicon clusters and indirectly demonstrated by the typical stick-and-slip behavior measured in FFM experiments independently from the chosen tip/substrate pairs.^52^

The critical role of surface passivation by hydrogen or by environmental molecules, such as water molecules, for achieving low friction coefficients has been highlighted by different experimental works, both for diamond and DLC films.^53−55^ Static first-principles calculations have quantified this effect on the ideal interfacial shear strength^9,56−58^ and *ab initio* MD simulations allowed us to monitor the tribochemical processes that lead to the diamond surface passivation by water during sliding.^4^ As a further step, we are now able to assess kinetic friction coefficients using QMGF MD simulations thanks to the capability to provide proper control of temperature, mechanical stresses, and energy dissipation in nonequilibrium conditions.

We further reduce the H coverage by considering a passivation of 25% and an H-free interface. In the former case, the sliding motion occurs only in the first stages of the simulations but then the slabs interlock due to the formation of chemical bonds across the interface, which are not broken by the applied lateral force. On the contrary, in the clean interface, the motion occurs with no interlocking. In Figure 6, a snapshot of the system acquired during the simulation reveals that a graphitization of the surfaces is taking place due to a partial rehybridization of the carbon surface bonds from sp^3^ to sp^2^. This determines the formation of interfacial graphene layers, which become almost detached from the diamond slabs.

The graphitization mechanism of carbon films induced by sliding has been observed experimentally,^59−61^ and by MD simulations,^9,62^ and it was related to the ultralow friction coefficient of diamond. The realistic simulations here performed clarify that the condition to achieve an ultralow friction coefficient is to make the surface–surface interaction change from chemical to physical. This condition can be realized either by increasing the level of passivation above a limiting value where the Pauli repulsion makes the surface separation high enough to inhibit the formation of bonds across the interface or by decreasing the passivation below a threshold value, where the surface graphitization takes place.

To evaluate the effects of the elastic properties of the semi-infinite bulks on the sliding dynamics and friction coefficients, we compare the results of QMGF MD with those of QM MD, obtained by decoupling the atomistic slabs from the bath of harmonic oscillators. Figure 7 shows the vertical separations of the diamond surfaces during the QM MD (a) and QMGF MD (b) simulations, where the same initial relative position of the two surfaces, external load, and shear are considered. In the absence of a proper description of the inertia and elasticity of the semi-infinite bulks through the GF MD, we observe a marked bumping of the surfaces. The bumping oscillations fade away quickly in the case of complete superficial passivation while they persist for 75 and 50% cases. While the average values of the surface separations are similar for the two kinds of simulations, the lack of contact between the surfaces after each bouncing event produces large system accelerations in the QM MD simulations and makes any quantitative estimate of frictional parameters absolutely not meaningful. This would have been even more evident if a model of the interface including roughness was used.

As a final point regarding the method, this is essential for capturing the response of a semi-infinite bulk along the direction normal to the interface. However, the computational cost required by ab initio simulations is not feasible for noncommensurate systems where large cells of simulations along longitudinal directions are required.

## IV Conclusions

Classical tribology was developed in the context of mechanical engineering, where friction forces are predicted on the basis of analytical models of contact mechanics. With the advent of nanotribology, it became possible to probe the tribological behavior of a single nano-asperity and, thanks to the increased power of supercomputers, reproduce it with fully atomistic models. It was then highlighted the importance of taking into account the atomic-scale roughness of the surfaces in contact.^63^ Then, the need to go beyond the atomistic description and consider also the electrons at the nano-asperity contacts emerged in the context of tribochemistry. Tight-binding MD and then more accurate, but computationally expensive, *ab initio* MD were introduced in the field of computational tribology to overcome the limited reliability of force fields in describing stress-assisted reactions (a comprehensive review on computational tribochemistry can be found here^7^).

All of the above-described approaches suffer from the limitation of using slabs of finite thickness, thus the energy introduced in the system through the application of external forces is “artificially” removed by thermostats that mimic the effects of the thermal bath consisting in the real systems of the infinite degrees of freedom of the bulk. This approximation makes any estimation of energy dissipation nonsense and prevents a full understanding of the interplay of adhesive and phononic contribution to fiction. To overcome these limitations, we developed a multiscale method, the QMGF method, that links *ab initio* to Green’s function MD.

We applied it to calculate the kinetic friction coefficient of two semi-infinite diamond bulks in contact, obtaining results in close agreement with experiments. We found that the friction coefficient, friction mechanisms, and interface morphology strongly depend on the degree of passivation of the diamond surfaces. We observe a superlubric regime at high H coverages, while below a threshold coverage, covalent bonds are established across the interface that causes the surface interlocking. This regime persists until the concentration of adsorbates becomes low enough to allow for a shear-induced change of hybridization of the surface carbon atoms from sp^3^ to sp^2^. Thanks to surface graphitization, the sliding motion is recovered. Our results indicate that this phenomenon can occur only when passivating species are almost absent from the interface; indeed, we observed graphitization for a clean diamond interface.

The above results point to the great potentiality of the QMGF method to provide highly accurate insights into interface phenomena in nonequilibrium conditions. This method may open the way to the investigation of other multiscale phenomena, where the infinite number of the bulk degrees of freedom, usually neglected in *ab initio* MD, is key in determining the system response to an external stimulus.

## Supporting Information Available

Application example of the fast convolution method (PDF)
