# Supplementary material for: Accurate Multiscale Simulation of Frictional Interfaces by Quantum Mechanics/Green’s Function Molecular Dynamics
Source: J Chem Theory Comput. 2023 Jul 11;19(15):5176–88. doi: 10.1021/acs.jctc.3c00295 (PMC10413868; doi:10.1021/acs.jctc.3c00295)
Supplement: Supplementary file 1 — ct3c00295_si_001.pdf [file ct3c00295_si_001.pdf]

Supplementary Information
Accurate multiscale simulation of frictional interfaces by Quantum Mechanics / Green's Function molecular dynamics

Seiji Kajita,[1,*] Alberto Pacini,[2] Gabriele Losi,[3] Nobuaki Kikkawa,[1] and Maria Clelia Righi[2,†]

[1] *Toyota Central R&D Labs., Inc., 41-1, Yokomichi, Nagakute, Aichi, 480-1192, Japan*
[2] *Department of Physics and Astronomy, University of Bologna, 40127 Bologna, Italy*
[3] *Department of Physics, Mathematics and Informatics, University of Modena and Reggio Emilia, 41125 Modena, Italy*

I. EXAMPLE OF FAST CONVOLUTION STEPS

The fast convolution is an elegant technique but could be somehow difficult to understand. We illustrate this procedure by an example in the case of $B = 2$ (this appendix is provided by referring to an excellent review [1] of this method). The time intervals are $I_0 = [0, h], I_1 = [h, 3h], I_2 = [2h, 7h], I_3 = [4h, 15h], \cdots$, where h is the single time step. The whole integral range $[0, t_{n+1}]$ is divided by I_l as shown in Fig. 1. The formula in the main text can be written as

$$\mathbf{y}'_{k+1} = \mathbf{y}'_k + \frac{e^{zh}-1}{zh}\left(zh\mathbf{y}'_k + h\mathbf{f}_k + h\frac{\mathbf{f}_{k+1}-\mathbf{f}_k}{zh}\right) - h\frac{\mathbf{f}_{k+1}-\mathbf{f}_k}{zh}, \tag{1}$$

$$\int_{t_n}^{t_{n+1}} A(t_{n+1}-\tau)\mathbf{f}(\tau)d\tau \sim \Phi_1\mathbf{f}(t_n) + \Phi_2\frac{\mathbf{f}(t_{n+1})-\mathbf{f}(t_n)}{h}, \tag{2}$$

$$\Phi_1 = \sum_{j=-N}^{N}\omega_j^0\frac{A(z_j^0)}{z_j^0}e^{z_j^0 h}, \quad \Phi_2 = \sum_{j=-N}^{N}\omega_j^0\frac{A(z_j^0)}{(z_j^0)^2}e^{z_j^0 h},$$

$$\int_0^{t_n} A(t_{n+1}-\tau)\mathbf{f}(\tau)d\tau \sim \sum_{l=1}^{L}\sum_{j=-N}^{N}\omega_j^l A(z_j^l)e^{z_j^l(t_{n+1}-\tau_{l-1})}\mathbf{y}'(\tau_{l-1},\tau_l,z_j^l), \tag{3}$$

where z, A, and $\mathbf{f}_k$ are the coordinate in the complex space, the Green's function, and the time-discretized force, respectively. The parameters L, N, and ω_j^l indicate the numbers of the integral paths, the discretization of the paths, and the coefficient of the integral, respectively (see the main text for detailed definitions).

Numerical steps of the time evolution are:

1. The first time step is $n = 0, t_{n+1} = h, L = 0$ and $\tau_0 = 0$. Therefore, the convolution consists only of Eq. 2.

2. The second step is $n = 1, t_{n+1} = 2h, L = 1, \tau_0 = h$ and $\tau_1 = 0$. The convolution term $\int_h^{2h} A(2h-\tau)\mathbf{f}(\tau)d\tau$ is calculated by Eq. 2, and the rest is obtained by Eq. 3, as

$$\int_h^{2h} A(2h-\tau)\mathbf{f}(\tau)d\tau \sim \sum_{-N}^{N}\omega_j^1 A(z_j^1)e^{z_j^1 h}\mathbf{y}'(h,0,z_j^1).$$

According to Eq. 1, $\mathbf{y}'(h,0,z_j^1)$ is derived from values of $\mathbf{f}(h)$ and $\mathbf{f}(0)$.

$$\mathbf{f}(h),\mathbf{f}(0) \to \mathbf{y}'(h,0,z_j^l) \quad l \geq 1$$

An important point is that here we save $\mathbf{y}'(h,0,z_j^l)$ values for all of the l contours in the memory. The force $\mathbf{f}(0)$ is not necessary to be saved.

* fine-controller@mosk.tytlabs.co.jp
† clelia.righi@unibo.it

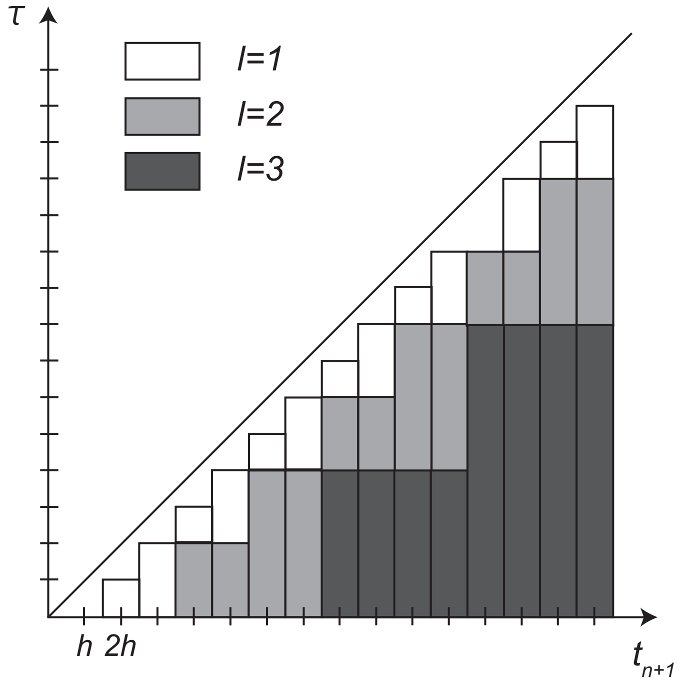

FIG. 1. Schematic image of the divided intervals of the fast convolution in the case of $B = 2$. Gray scales in the rectangles indicate the mTILT performed by the corresponding l contour.

3. The third step is $n = 2, t_{n+1} = 3h, L = 1, \tau_0 = 2h$ and $\tau_1 = 0$. As for the second step, $\int_{2h}^{3h} A(3h-\tau)\mathbf{f}(\tau)d\tau$ is calculated by Eq. 2, and the rest is obtained by Eq 3, as

$$\int_0^{2h} A(3h-\tau)\mathbf{f}(\tau)d\tau \sim \sum_{-N}^{N} \omega_j^1 A(z_j^1) e^{z_j^1 h} \mathbf{y}'(2h, 0, z_j^1).$$

$\mathbf{y}'(2h, 0, z_j^l)$ values are also calculated from the force terms and $\mathbf{y}'(h, 0, z_j^l)$ via Eq. 1 and saved.

$$\mathbf{y}'(h, 0, z_j^l), \mathbf{f}(2h), \mathbf{f}(h) \to \mathbf{y}'(h, 0, z_j^l) \quad l \geq 1$$

The force $\mathbf{f}(h)$ is not necessary to be saved any more.

4. The next step is $n = 3, t_{n+1} = 4h, L = 2, \tau_0 = 3h, \tau_1 = h$ and $\tau_2 = 0$. The first term $\int_{2h}^{3h} A(3h-\tau)\mathbf{f}(\tau)d\tau$ is calculated by Eq. 2. Because now L becomes 2, the rest convolution consists of the contributions of $l = 1$ and $l = 2$,

$$\int_{2h}^{3h} A(4h-\tau)\mathbf{f}(\tau)d\tau \sim \sum_{-N}^{N} \omega_j^1 A(z_j^1) e^{z_j^1 h} \mathbf{y}'(3h, 2h, z_j^1).$$

$$\int_0^{2h} A(4h-\tau)\mathbf{f}(\tau)d\tau \sim \sum_{-N}^{N} \omega_j^2 A(z_j^2) e^{2z_j^2 h} \mathbf{y}'(2h, 0, z_j^2),$$

Here we can import the $\mathbf{y}'(2h, 0, z_j^2)$ value that was saved at the previous step. Note that the superscript indicates the l index, not to the power of the corresponding variable. The $\mathbf{y}'(3h, 2h, z_j^1)$ term is calculated by Eq. 1,

$$\mathbf{f}(3h), \mathbf{f}(2h) \to \mathbf{y}'(3h, 2h, z_j^1).$$

We also proceed the time step forward to the saved $\mathbf{y}'$ values as

$$\mathbf{y}'(2h, 0, z_j^l), \mathbf{f}(3h), \mathbf{f}(2h) \to \mathbf{y}'(3h, 0, z_j^l). \quad l \geq 2$$

The force $\mathbf{f}(2h)$ is not necessary to be saved.

5. In the same manner, we calculate the convolution. Especially, the $\mathbf{y}'$ value we need is

$$\mathbf{y}'(3h, 2h, z_j^1), \mathbf{f}(4h), \mathbf{f}(3h) \rightarrow \mathbf{y}'(4h, 2h, z_j^1).$$

Then, the saved $\mathbf{y}'$ values are proceeded by one time step in order to prepare the subsequent time evolution.

$$\mathbf{y}'(3h, 0, z_j^l), \mathbf{f}(4h), \mathbf{f}(3h) \rightarrow \mathbf{y}'(4h, 0, z_j^l). \quad l \geq 2$$

As shown above, we do not need to save all the history of the force during the dynamic simulation. Instead, the stored $\mathbf{y}'$ values are updated at each time step, and the updated values are used to perform the convolution employing mTILT. This procedure allows us to decrease the memory allocation and simulation time dramatically.

[1] Prete, I. D. Efficient numerical methods for Volterra integral equations of Hammerstein type, Doctoral thesis. Ph.D. thesis, Universita degli Studi di Napoli Federico II, 2006.
